# Supplementary material for: Moral decision-making is altered in patients with schizophrenia
Source: PLoS One. 2025 May 2;20(5):e0322614. doi: 10.1371/journal.pone.0322614 (PMC12047836; doi:10.1371/journal.pone.0322614)
Supplement: Attachment — Submitted filename: Academic Editor comments 12-26-24.docx [file pone.0322614.s001.docx]

Thank you for your scholarly contribution to the topic area of schizophrenia. I'd also like to thank the authors for choosing PLOS ONE to publish your findings from this study. My review comments and comments from other experts in this topic area are provided below. Please review these comments and I suggest address them and resubmit your manuscript. Your timely response would help this study be published and will make it accessible to interested readers across the world. I look forward to reviewing your revised manuscript. I wish you good luck with your future endeavors.

Editor comments:

On page 3, materials and methods - "Participants" section, please provide inclusion criteria for study participants.

On page 4, under "measures" section: Please provide psychometric properties for "the moral questionnaire" and for the "GHQ-12".

On page 5, under "Demographic characteristics", please consider changing "overall mean age" to "overall mean age in years"

On page 5, under "Demographic characteristics", please clarify why the standard deviation was greater than the mean or is it a typo? ".... with 2.16 ± 2.92 numbers of hospitalisation."

Please provide if there were any dropouts or if any of the items had a non-response.

One of the limitations of this study could be a relatively low sample size which could have affected the power to detect subtle differences between the two groups. If you agree with this assessment please acknowledge this in the limitations section on page 8.

On page 3, the authors state "Moreover, as the purpose of our study, the focus on everyday moral dilemmas will provide precious insights into the functional implications of these deficits, ultimately contributing to the development of targeted interventions to enhance social functioning in patients with schizophrenia." However, I could not see any reasonable discussion on "insights into the functional implications of these deficits" in the discussion section. Please address this in your next revision.
