# Supplementary material for: Moral decision-making is altered in patients with schizophrenia
Source: PLoS One. 2025 May 2;20(5):e0322614. doi: 10.1371/journal.pone.0322614 (PMC12047836; doi:10.1371/journal.pone.0322614)
Supplement: Attachment — Submitted filename: Response to reviewers.docx [file pone.0322614.s002.docx]

We greatly thank the respected editors and reviewers for their valuable time and comments on our manuscript. We have thoroughly considered their constructive comments and amended the manuscript accordingly.

In short, discussion and last paragraph of introduction have been amended according to the comments of respected editors and reviewers. The discussion now contains sections on the interplay of cognition and moral reasoning, and functional implications and interventions. Methods section has been edited to more clearly present patient inclusion and analysis rationale. Psychometric properties of utilized questionnaires have also been added. Lastly, a new table containing patient demographics has been added to the results, and the previous table has been amended to contain more detailed presentations of results. The respective change regarding each point is tracked in the uploaded revised manuscript.

We’d also like to note that the affiliation for “Atiye Sarabi-Jamab” and “Fatemeh Sadat Mirfazeli” have been updated. We apologize for any inconvenience this might cause. We would like to mention that Fatemeh Sadat Mirfazeli is the Co-corresponding author with Dr. Seyed Vahid Shariat.

Thank you very much for your assistance in enhancing our manuscript. Below are our point-to-point responses to the comments:

**Academic Editor Comments:**

***1.*** *On page 3, in the materials and methods "Participants" section, please provide inclusion criteria for the study participants.*

Author response:

Thank you for your comment to enhance the quality of the manuscript. The methods section regarding participant inclusion has been rewritten to present the process more clearly for patients and control subjects.

Manuscript has been changed as follows:

“This cross-sectional study recruited 64 participants (32 patients, 32 control subjects) from clinics of university-affiliated hospitals between June 1^st^ and November 30^th^ 2021.

Patients from two different outpatient psychiatry clinics who were diagnosed with schizophrenia according to the Diagnostic and Statistical Manual of Mental Disorders (DSM)-5, as established by an attending psychiatrist, were included in the study.”

“Control subjects were recruited from all outpatient clinics of university-affiliated hospitals, excluding psychiatry, psychology, neurology, and neurosurgery clinics. Subjects with a General Health Questionnaire (GHQ-12) score of less than 23 were included as the control group.”

***2.*** *On page 4, under "measures" section, Please provide psychometric properties for "the moral questionnaire" and for the "GHQ-12".*

Author response:

Thank you for your comment. The methods section now contains the measures for reliability of both questionnaires.

“Due to the length of the original questionnaire, a shorter version, containing 40 vignettes, has been translated, abbreviated, and validated in the Persian language by Yazdanpanah et al [14], which was utilized in this study. The internal consistency of the shortened vignettes in the Iranian culture, measured by Cronbach’s alpha, was 0.895. The Cronbach’s alpha measurement of the original 150-vignette questionnaire was 0.96 and it was shown to be consistent with Iranian culture [14].”

“The mental health of control subjects was measured by a Persian-translated and validated version of GHQ-12. This questionnaire is considered valid and reliable across various settings and cultures, such as Iran (Cronbach’s alpha measure: 0.87, Convergent validity: r = -.56, P<0.0001) [15], Finland (Cronbach’s alpha measure: 0.92), India (Cronbach’s alpha measure: 0.82), and South Korea (Cronbach’s alpha measure: 0.81) [16].”

***3.*** *On page 5, under "Demographic characteristics", please consider changing "overall mean age" to "overall mean age in years"*

Author response:

Thank you for your comment. A change has been made to the sentence accordingly.

***4.*** *On page 5, under "Demographic characteristics", please clarify why the standard deviation was greater than the mean or is it a typo? ".... with 2.16 ± 2.92 numbers of hospitalization."*

Author response:

Thank you for your meticulousness. Due to the data's non-normality, the SD was greater than the mean. To better represent the baseline characteristics, the data presentation has been changed to median for age and median [range] for disease duration and number of hospitalizations in the manuscript text.

***5.*** *Please provide if there were any dropouts or if any of the items had a non-response:*

Author response:

Thank you for your comment. There were no dropouts throughout the study, and participants responded to all items in the questionnaire. This information is now included in the methods section.

***6.*** *One of the limitations of this study could be a relatively low sample size which could have affected the power to detect subtle differences between the two groups. If you agree with this assessment please acknowledge this in the limitations section on page 8.*

Author response:

The sample size calculations were based on previous publications with comparable methodologies [7, 14, 36, 37] and the below formula, considering 95% confidence level and a 5% margin of error which counts a sample size of around 64, 32 control groups, and 32 patients.

$$n_{1}=\frac{{(\sigma_{1}^{2}+\frac{\sigma_{2}^{2}}{K})(Z_{1-\frac{\alpha}{2}}+Z_{1-\beta})}^{2}}{\Delta^{2}}$$

$$k=\frac{n_{2}}{n_{1}}=1$$

However, the relatively low sample size could have affected the power of our analysis, and studies with larger sample sizes could detect any other possible subtle differences between the two groups. This limitation is mentioned in the limitations section of the manuscript.

Manuscript has been changed as follows:

“Although an accurate sample size calculation could not be made due to no available studies utilizing the same questionnaire in patients with schizophrenia, a conceivable sample size was calculated with 95% confidence level and a 5% margin of error as well as considering studies with comparable methodology [7, 14, 36, 37]. However, low sample size of this study could affect the power of analysis to detect subtle differences between the two groups.”

***7.*** *On page 3, the authors state "Moreover, as the purpose of our study, the focus on everyday moral dilemmas will provide precious insights into the functional implications of these deficits, ultimately contributing to the development of targeted interventions to enhance social functioning in patients with schizophrenia." However, I could not see any reasonable discussion on "insights into the functional implications of these deficits" in the discussion section. Please address this in your next revision.*

Author response:

Thank you for your comment. The discussion has been amended to further explain the possible functional implications and targeted interventions. The mentioned sentences of the introduction have also been slightly adjusted.

Manuscript has been changed as follows:

Introduction: “Moreover, the focus on everyday moral dilemmas with multi-aspect dimensions could provide precious insights into the functional implications of these deficits, ultimately contributing to the development of targeted interventions such as socio-cognitive rehabilitation to enhance social functioning in patients with schizophrenia [12].”

Discussion: “The observed alterations in evaluating the benefit and moral appropriateness of actions among patients with schizophrenia could have significant implications for real-life social functioning. These changes could lead to deficient social cognition and difficulty interpreting and understanding others intentions, resulting in strained personal and professional relationships [31, 32]. Recent meta-analyses have reported that social-cognitive training, alongside the basic aspects of social cognition, can remediate more complex social-cognitive processes such as social perception and mental state attribution [12, 33]. A combination of targeted interventions, such as cognitive behavioral therapy, social cognition and social skill training, and practice weighing self- and other-focused consequences in decisions, could improve perspective-taking and help restructure maladaptive thought patterns in patients [34, 35].”

**Journal requirements:**

***1.*** *Please ensure that your manuscript meets PLOS ONE's style requirements.*

Author response:

The manuscript has been edited according to journal requirements.

***2.*** *Please describe in your methods section how capacity to provide consent was determined for the participants in this study. Please also state whether your ethics committee or IRB approved this consent procedure. If you did not assess capacity to consent please briefly outline why this was not necessary in this case.*

Author response:

Thank you for your comment. The methods section, under “ethical considerations”, now explains how capacity to provide consent was determined.

Additionally, the ethics committee of IUMS approved this procedure and all the steps of the study.

Manuscript has been changed as follows:

“The capacity to provide consent was determined through the clinical judgment of the attending physician, ensuring participants understood the study’s purpose and their voluntary participation. The patients who had the capacity for consent were included in the study. The ethics committee of the Iran University of Medical Sciences provided approval for all study procedures.”

***3.*** *In the online submission form you indicate that your data is not available for proprietary reasons and have provided a contact point for accessing this data. Please note that your current contact point is a co-author on this manuscript. According to our Data Policy, the contact point must not be an author on the manuscript and must be an institutional contact, ideally not an individual. Please revise your data statement to a non-author institutional point of contact, such as a data access or ethics committee, and send this to us via return email. Please also include contact information for the third party organization, and please include the full citation of where the data can be found.*

Author response:

The contact point for accessing data has been changed to the National Brain Centre and the manuscript has been adjusted accordingly.

***4.*** *PLOS requires an ORCID iD for the corresponding author in Editorial Manager on papers submitted after December 6th, 2016. Please ensure that you have an ORCID iD and that it is validated in Editorial Manager. To do this, go to ‘Update my Information’ (in the upper left-hand corner of the main menu), and click on the Fetch/Validate link next to the ORCID field. This will take you to the ORCID site and allow you to create a new iD or authenticate a pre-existing iD in Editorial Manager.*

Author response:

The ORCID iD for the corresponding authors has been added to the profile page in the editorial manager.

***5.*** *We note that you have referenced (Escobedo JR. Investigating moral events: characterization and structure of autobiographical moral memories. (Unpublished dissertation), Pasadena, CA: California Institute of Technology. 2009.) which has currently not yet been accepted for publication. Please remove this from your References and amend this to state in the body of your manuscript: (ie “Bewick et al. [Unpublished]”) as detailed online in our guide for authors* [*http://journals.plos.org/plosone/s/submission-guidelines#loc-reference-style*](http://journals.plos.org/plosone/s/submission-guidelines#loc-reference-style)

Author response:

Thank you for your meticulousness. Considering that the mentioned “Escobedo JR et. al.” manuscript is a published dissertation with an assigned DOI, this citation is now edited and entered as a “Master's thesis or doctoral dissertation” according to the instructions provided in the PLOS ONE journal webpage.

Additionally, the manuscript now uses the PLoS referencing style suggested in the journal webpage.

**Review Comments to the Author:**

**Reviewer #1:**

We greatly thank the respected reviewers for their valuable time and comments on our manuscript. We have thoroughly considered their constructive comments and amended the manuscript accordingly.

***1.*** *Statistical Presentation:
- Table 1 would benefit from including descriptive statistics (means, standard deviations) for both groups.
- Addition of effect sizes and confidence intervals would help readers better understand the magnitude of differences between groups*

Author response:

Thank you for your valuable comments. In line with adding a new table, previous table one has been updated to table two and now contains descriptive statistics (mean/SD and median/range) for responses of both groups. In addition, the Generalized Ets Squared (GES) values have been added to the table, as a representative of effect size.

Considering the already crowded table 2, we refrained from reporting additional values listed in the table below, including the difference between control subjects minus patients with 95% CI. These values could be reported in the manuscript if the respected reviewer deems it necessary.

| **Dimension** | **Moral Vignettes** | | | | **Immoral Vignettes** | | | | |
| --- | --- | --- | --- | --- | --- | --- | --- | --- | --- |
|  | generalized eta squared (ges) | diff(control subjects – patients with schizophrenia) | 95% confidence interval* | p-adj | generalized eta squared (ges) | diff(control subjects - patients with schizophrenia) | 95% confidence interval* | p-adj |  |
| **Emotional intensity** | 0.03 | -0.44 | -1.08, 0.19 | 0.17 | 0.026 | -0.39 | -1.00  0.22 | 0.21 |  |
| **Emotional aversion** | 0.045 | -0.43 | -0.95, 0.08 | 0.94 | 0.04 | -0.38 | -0.85  0.09 | 0.11 |  |
| **Harm** | 0.034 | -0.37 | -0.87, 0.13 | 0.15 | 0.029 | 0.22 | -0.10, 0.55 | 0.18 |  |
| **Self-benefit** | 0.129 | -0.70 | -1.17, -0.28 | 0.004 | 0.072 | 0.59 | 0.05, 1.13 | 0.03 |  |
| **Other benefit** | 0.017 | -0.28 | -0.82, 0.28 | 0.30 | 0.0114 | -0.62 | -1.07, -0.18 | 0.00 |  |
| **Pre-mediation** | 0.00011 | 0.02 | -0.55, 0.59 | 0.94 | 0.074 | 0.62 | 0.06, 1.17 | 0.03 |  |
| **Legality** | 0.013 | 0.21 | -0.27, 0.70 | 0.38 | 0.048 | -0.36 | -0.76, 0.05 | 0.08 |  |
| **Social norm violation** | 0.034 | -0.34 | -0.79, 0.12 | 0.14 | 0.06 | -0.51 | -1.02, 0.00 | 0.05 |  |
| **Socialness** | 0.046 | -0.53 | -1.13, 0.08 | 0.09 | 0.007 | -0.19 | -0.77, 0.39 | 0.51 |  |
| **Frequency** | 0.005 | 0.15 | -0.39, 0.66 | 0.56 | 0.027 | 0.42 | -0.23, 1.07 | 0.19 |  |
| **Personal familiarity** | 0.052 | 0.47 | -0.04, 0.98 | 0.07 | 0.065 | -0.41 | -0.81, -0.01 | 0.04 |  |
| **General familiarity** | 0.046 | 0.45 | -0.07, 0.97 | 0.09 | 0.027 | 0.38 | -0.20, 0.96 | 0.19 |  |
| **Moral appropriateness** | 0.003 | 0.09 | -0.33, 0.51 | 0.65 | 0.214 | -0.74 | -1.09, -0.38 | 0.00 |  |

***2.*** *Intervention Context:
- The discussion would benefit from better contextualizing the findings within the broader framework of interventions mentioned in the introduction
- Further elaboration on how the observed alterations in moral decision-making relate to real-life social functioning challenges
- Clearer implications of these findings for developing targeted interventions to enhance social functioning in patients with schizophrenia:*

Author response:

Thank you for your precious comment on enhancing the quality of our work. A paragraph has been added to the discussion to elaborate on the context of real-life functioning and implications for interventions.

Manuscript has been changed as follows:

“The observed alterations in evaluating the benefit and moral appropriateness of actions among patients with schizophrenia could have significant implications for real-life social functioning. These changes could lead to deficient social cognition and difficulty interpreting and understanding others intentions, resulting in strained personal and professional relationships [31, 32]. Recent meta-analyses have reported that social-cognitive training, alongside the basic aspects of social cognition, can remediate more complex social-cognitive processes such as social perception and mental state attribution [12, 33]. A combination of targeted interventions, such as cognitive behavioral therapy, social cognition and social skill training, and practice weighing self- and other-focused consequences in decisions, could improve perspective-taking and help restructure maladaptive thought patterns in patients [34, 35].”

***3.*** *Intelligence and Cognitive Function:
- A more detailed discussion of how cognitive function might influence moral decision-making
- Clarification of whether and how intelligence was assessed beyond basic screening: methods or limitation or discussion?
- Discussion of the relationship between general intelligence and moral reasoning capacity
- How cognitive function might mediate the observed differences between groups*

Author response:

Thank you for your precious comment on enhancing the quality of our work. The discussion has been amended to include a paragraph on the relationship between cognitive function, intelligence, and moral decision-making.

Manuscript has been changed as follows:

“Cognitive processes play a pivotal role in moral decision-making and facilitate the evaluation of ethical dilemmas, integration of social and emotional considerations, and balancing interests. Impaired executive function, social cognition, and cognitive flexibility could significantly influence moral reasoning in patients [21, 23, 24]. Furthermore, theory of mind deficits, which affect the ability to understand others' perspectives and intentions, could mediate the observed differences in how patients and control subjects assess vignettes [21, 22]. Future studies exploring both theory of mind and everyday moral decision-making in patients with schizophrenia could shed light on how the interaction of benefit and theory of mind could shape moral judgment.

General intelligence has been argued to mediate moral decision-making [25] and affect patients sociomoral reasoning capabilities [26]. Nonetheless, it has been demonstrated that patients with preserved intellectual functioning have similar cognitive deficits to patients with compromised general intelligence, indicating that impaired cognitive processes are inherent characteristics of schizophrenia regardless of intelligence levels [27]. Our study assessed educational degree as a measure of baseline cognitive ability and intelligence, and there were no significant differences among the groups.”

**Reviewer #2:**

We greatly thank the respected reviewers for their valuable time and comments on our manuscript. We have thoroughly considered their constructive comments and amended the manuscript accordingly.

***1.*** *According to the journal’s submission guidelines, the introduction should include a brief statement of the overall aim of the work, along with a comment on whether that aim was achieved.*

Author response:

Thank you for your valuable comment. The introduction has been changed to include a brief statement on the overall aim of the work and the final achieved aim.

Manuscript has been changed as follows:

“This study investigates everyday moral decision-making in patients with schizophrenia and demonstrates that most aspects of moral decision-making remain intact in these patients. The observed differences seem to be limited to benefit assessment and evaluation of moral appropriateness.”

***2.*** *In the description of the task, it is not explained how the individual scores were measured. Was this a median score computed from the individual's responses?*

Author response:

In our analyses, the averages of participants’ responses in each dimension were compared between groups based on two categories (moral and immoral). The methods section, under statistical analysis, has been adjusted to more clearly state this information.

*3. The description of the statistical analysis needs revision, as it is currently not clear.*

Author response:

Thank you for your valuable comments on enhancing our manuscript.

The statistical analysis section has been rewritten to more clearly present the analytic approach. We hope that this change meets the reviewers' standards.

Manuscript has been changed as follows:

“Descriptive statistics, chi-squared test, and independent sample t-test were used to analyze the data. The significance level was considered as 0.05. Data with normal distribution are presented as mean ± SD, otherwise, data and demographic characteristics are reported as median (IQR) and median [range]. ANOVA test was used to analyze responses to the questionnaire and their relationship to other variables and check for statistically significant differences between the two groups on participants’ averaged responses. The averages of participants’ responses to each dimension were compared between groups based on two categories (moral and immoral). As multiple hypotheses were tested simultaneously, the most possible type I errors were considered as the 13 dimensions in the questionnaire. Accordingly, the significance level of ANOVA test was set as 0.0038. The Bonferroni adjustment was used to correct the experiment-wise error rate. All statistical analyses were performed using R (version 3.6.1; R Core Team, 2019) in RStudio (RStudio- 1.2.5001).”

***4.*** *Demographic characteristics (such as gender and education) are not described. Please add this information to the text or present it in a table.*

Author response:

Thank you for your valued comment. A new table presenting the demographic characteristics of participants has been added to the manuscript (now Table 1 in the manuscript).

**Table 1.** Demographic characteristics of participants

| **Variable** | **Patients with schizophrenia (N=32)** | **Healthy subjects (N=32)** | **P value** |
| --- | --- | --- | --- |
| **Age** | 38 (9.5) | 36.5 (9.5) | 0.652 |
| **Gender (Male/Female)** | 23/9 | 23/9 | 1 |
| **Education level** | 59.4% diploma  40.6% bachelors | 40.6% diploma  59.4% bachelors | 0.133 |
| **Length of disease** | 11 [1-39] | N/A | - |
| **Number of hospitalization** | 1.5 [0-15] | N/A | - |

Data presented as median (IQR), and median [range].

N/A: Not applicable,

***5.*** *In Table 1, I recommend presenting the F-values first, followed by the p-values.*

Author response:

Thank you for your precious comment. This table now presents F values before p values.

**Table 2.** Differences between patients with schizophrenia and control subjects in moral and immoral vignettes

| **Dimension** | **Moral Vignettes** | | | | | **Immoral Vignettes** | | | | |
| --- | --- | --- | --- | --- | --- | --- | --- | --- | --- | --- |
|  | Patients responses* | Control subjects responses* | F test | P value | GES** | Patients responses* | Control subjects responses* | F test | P value | GES** |
| **Emotional intensity** | 4.68(1.42) | 4.23(1.11) | 1.93 | 0.17 | 0.03 | 4.17(1.21) | 3.78(1.22) | 3.857 | 0.054 | 0.026 |
| **Emotional aversion** | 3.76(1.04) | 3.32(1.02) | 2.89 | 0.094 | 0.045 | 4.7(0.86) | 4.39(1.03) | 3.405 | 0.07 | 0.04 |
| **Harm** | 2.86(1.18) | 2.48(0.78) | 2.165 | 0.146 | 0.034 | 4.56(0.74) | 4.80(0.54) | 0.718 | 0.4 | 0.029 |
| **Self-benefit** | 3.01(0.93) | 2.12[1-5] | 0.167 | 0.004 | 0.129 | 3.79(1.17) | 4.52[1.5-6] | 5.557 | 0.022 | 0.072 |
| **Other-benefit** | 4.09(0.92) | 3.81(1.21) | 1.09 | 0.301 | 0.017 | 2.69[1.22-4.47] | 1.98[1.12-3.28] | 9.7 | 0.003 | 0.0114 |
| **Pre-mediation** | 3.03(1.25) | 3.06(1.04) | 0.007 | 0.935 | 0.00011 | 3.95(1.36) | 4.57(0.78) | 4.134 | 0.046 | 0.074 |
| **Legality** | 5.08(0.98) | 5.30(0.94) | 0.797 | 0.376 | 0.013 | 3.41(0.80) | 2.84[1.53-5.22] | 4.289 | 0.043 | 0.048 |
| **Social norm violation** | 5.08(0.98) | 4.74(0.82) | 2.205 | 0.143 | 0.034 | 3.12(0.87) | 2.23[1.06-5.94] | 4.204 | 0.045 | 0.06 |
| **Socialness** | 5.25(0.98) | 4.94[2.25-6.63] | 2.965 | 0.09 | 0.046 | 4.61(1.16) | 4.42(1.17) | 0.705 | 0.404 | 0.007 |
| **Frequency** | 4.14(1.25) | 4.28(0.71) | 0.342 | 0.561 | 0.005 | 3.98(1.42) | 4.40(1.18) | 1.512 | 0.224 | 0.027 |
| **Personal familiarity** | 3.02(1.17) | 3.49(0.83) | 3.435 | 0.069 | 0.052 | 2.016[1.12-4.5] | 1.97(0.46) | 3.694 | 0.059 | 0.065 |
| **General familiarity** | 4.03(1.28) | 4.48(0.73) | 2.974 | 0.09 | 0.046 | 4.07(1.37) | 4.45(0.93) | 1.151 | 0.288 | 0.027 |
| **Moral appropriateness** | 5.5(1.02) | 5.64(0.59) | 0.201 | 0.656 | 0.003 | 2.97(0.85) | 2.23(-0.56) | 15.5 | 0.0002 | 0.214 |

Bonferroni correction: alpha=0.0038

*Data presented as mean (SD) or median [range]

** Generalized Eta Squared

We once again thank the editors and reviewers for their time and valuable input and hope that the revisions to the manuscript meet their standards.
