# Supplementary material for: Moral decision-making is altered in patients with schizophrenia
Source: PLoS One. 2025 May 2;20(5):e0322614. doi: 10.1371/journal.pone.0322614 (PMC12047836; doi:10.1371/journal.pone.0322614)
Supplement: Attachment — Submitted filename: Academic Editor comments 03-10-25.docx [file pone.0322614.s003.docx]

Thank you for your scholarly contribution to the topic area of schizophrenia. I'd also like to thank the authors for choosing PLOS ONE to publish your findings from this study. My review comments are provided below. Please review these comments and I suggest address them and resubmit your manuscript. Your timely response would help this study be published and will make it accessible to interested readers across the world. I look forward to reviewing your revised manuscript. I wish you good luck with your future endeavors.

Academic Editor comments:

Abstract: Results section: Please provide p values in parenthesis at the end of first two statements.

Line 89: Participants: Did you consider age of participants while recruiting study participants? If so mention this in the inclusion / exclusion criteria.

Line 104: What was the criteria used to define "alcohol addiction" and how did you screen participants for "alcohol addiction". Please explain and include in this section.
