# Supplementary material for: Moral decision-making is altered in patients with schizophrenia
Source: PLoS One. 2025 May 2;20(5):e0322614. doi: 10.1371/journal.pone.0322614 (PMC12047836; doi:10.1371/journal.pone.0322614)
Supplement: Attachment — Submitted filename: Response_to_Reviewers_auresp_2.docx [file pone.0322614.s004.docx]

Thank you very much for taking the time to revise and improve our manuscript. Below are our responses to your valuable comments:

**Academic Editor Comments:**

***1.*** *Abstract: Results section: Please provide p values in parenthesis at the end of first two statements.*

Author response:

Thank you for your comment. Abstract now contains the requested p values.

***2.*** *Line 89: Participants: Did you consider age of participants while recruiting study participants? If so mention this in the inclusion / exclusion criteria.*

Author response:

Thank you for your comment. Participants younger than 18 and older than 65 years were excluded from the study. This information is now mentioned in line 103.

***3.*** *Line 104: What was the criteria used to define "alcohol addiction" and how did you screen participants for "alcohol addiction". Please explain and include in this section.*

Author response:

Thank you for your comment. Alcohol use in patients was judged based on DSM-5 criteria for alcohol use disorder. “Alcohol addiction” has been altered to “alcohol use disorder” for more precise definition.

The manuscript now mentions that “Alcohol use disorder was assessed through interview based on DSM-5 criteria.”.

**Journal Requirements:**

*Please review your reference list to ensure that it is complete and correct. If you have cited papers that have been retracted, please include the rationale for doing so in the manuscript text, or remove these references and replace them with relevant current references. Any changes to the reference list should be mentioned in the rebuttal letter that accompanies your revised manuscript. If you need to cite a retracted article, indicate the article’s retracted status in the References list and also include a citation and full reference for the retraction notice.*

Author response:

The reference list was reviewed to make sure all references match journal requirements. The PLoS style has been used for reference management.

The data availability statement has also been edited to explain the restrictions further, as requested by the respected editorial office.

We once again thank the editorial team for their attentive review of our manuscript and are at your disposal for any further changes that may be required.
